# Decrease in incidence of oral anticoagulant-related intracerebral hemorrhage over the past decade in the Netherlands

**DOI:** 10.1177/23969873211062011

**Published:** 2022-02-17

**Authors:** Michaël TJ Peeters, Florence Vroman, Tobien AHCML Schreuder, Robert J van Oostenbrugge, Julie Staals

**Affiliations:** 1 Department of Neurology, School for Cardiovascular Diseases (CARIM), 82246 Maastricht University Medical Center, the Netherlands; 2 Faculty of Health, Medicine and Life Sciences, 82246 Maastricht University Medical Center, the Netherlands; 3 Department of Neurology, 3802 Zuyderland Medical Center, the Netherlands

**Keywords:** Primary intracerebral hemorrhage, anticoagulants, epidemiology, incidence

## Abstract

**Background:**

Data on oral anticoagulant-related (OAC) intracerebral hemorrhage (ICH) incidence are scarce. Most studies on incidence time trends were performed before the introduction of Direct Oral Anticoagulants (DOACs). Between 2008 and 2018, the number of OAC-users in the Netherlands increased by 63%, with the number of DOAC-users almost equaling that of Vitamin K Antagonists (VKA)-users. We aimed to determine the recent total and OAC-related ICH incidence and assess changes over the last decade, including the effect of DOAC introduction.

**Methods:**

All adult non-traumatic ICH patients presenting in any of three hospitals in the enclosed region of South-Limburg, the Netherlands, were retrospectively included, during two 3-year time periods: 2007–2009 and 2017–2019. OAC-related ICH was defined as ICH in patients using VKAs or DOACs. We calculated the incidence rate ratio (IRR) between the two study periods.

**Results:**

In the 2007–2009 period, we registered 652 ICHs of whom 168 (25.8%) were OAC-related (all VKA). In the 2017–2019 period, we registered 522 ICHs, 121 (23.2%) were OAC-related (70 VKA and 51 DOAC). In 2007–2009, the annual incidence of total ICH and OAC-related ICH was 40.9 and 10.5 per 100,000 person-years, respectively, which decreased to 32.4 and 7.5 per 100,000 person-years in 2017–2019. The IRR for total ICH and OAC-related ICH was 0.67 (95%-CI: 0.60–0.75) and 0.58 (0.46–0.73), respectively.

**Conclusion:**

Both total ICH and OAC-related ICH incidence decreased over the past decade in South-Limburg, the Netherlands, despite the aging population and increasing number of OAC-users. The introduction of DOACs, and possibly an improved cardiovascular risk management and change in OAC prescription pattern, could explain these findings.

## Introduction

Intracerebral hemorrhage (ICH) is a feared and devastating complication of the use of oral anticoagulants (OAC) and is associated with high mortality and high morbidity.^1,2^ Until recently, Vitamin K antagonists (VKAs) were the single and most used OAC; however, since about 2010, Direct Oral Anticoagulants (DOACs) are available, and the number of DOAC users now exceeds that of VKA users in the Netherlands.^3^ Furthermore, DOACs reduce the risk of ICH about a half compared to VKAs.^4,5^

Recent data suggest a declining trend in stroke incidence, especially in ischemic stroke incidence.^6,7^ However, this remains unclear for the incidence of ICH. A Dutch population study noted a decrease in ICH incidence between 1998 and 2010, especially in patients younger than 75 years.^1^ However, the two largest studies to date reporting on ICH incidence outside of the Netherlands found stable ICH incidence rates.^8,9^ Several other studies on ICH incidence have been performed, and while some recent studies show a decrease in ICH incidence, others show a stable or even increased ICH incidence rate.^6,7,10–17^ The difference in investigated time-periods, patient selection, and study size, with several studies encompassing single institutional cohorts, could explain these diverse findings.

Data on incidence of OAC-related ICH are limited and mostly dating from before the introduction of DOACs, and generally show increasing OAC-related incidence trends.^10,18^ We found only two studies on OAC-related ICH incidence in the past decade, including the introduction of DOAC, which both show an increase in OAC-related ICH incidence rate in the past decade.^19,20^

We previously determined the incidence of OAC-related ICH in South-Limburg, the Netherlands, in 2007–2009.^21^ We now re-studied the incidence in the same population area. The aim of this study was to determine the recent annual total and OAC-related ICH incidence and to assess changes in incidence over the past decade, including the effect of the introduction of DOACs in the Netherlands.

## Methods

### Patient selection

**Figure 1.**
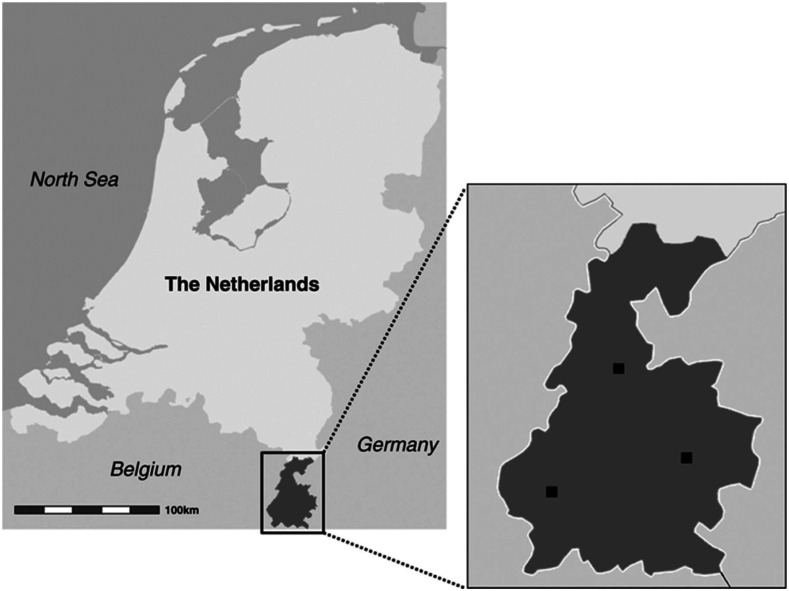
The Netherlands with South-Limburg enlarged. The participating hospitals are marked. Figure adjusted from Schols et al.^21^

We performed a multicenter, retrospective study over two 3-year time periods. The 2007–2009 cohort has been published before and the same catchment area and in- and exclusion criteria were used for both study periods.^21^ We included all consecutive patients ≥ 18 years presenting with a non-traumatic ICH at the emergency department or inpatient clinic in any of the three hospitals in the well-defined and bordered region of South-Limburg, the Netherlands (Figure 1), during two time periods: from January 2007 through December 2009 and from January 2017 through December 2019. We did not include 2020 in the study period because the COVID-19 pandemic might have influenced ICH referral and admittance policies. Recurrent ICHs were included, except for recurrences during the same hospital admission. Exclusion criteria were age < 18 years, patients living outside the postal code area of South-Limburg, traumatic ICH, non-parenchymatous intracranial hematoma (e.g., subdural, epidural, subarachnoidal, or primary intraventricular hemorrhage without visible parenchymatous involvement), ischemic stroke with hemorrhagic transformation as well as ICH associated with a brain tumor or encephalitis.

### Data collection

For clinical and radiological data, we used the same definitions for both time periods.^21^ We recorded age, sex, previous ICH, hypertension (defined as treatment with antihypertensives or hypertension in medical history), diabetes mellitus (defined as treatment with antidiabetics or known diabetes mellitus in medical history), hypercholesterolemia (defined as treatment with a statin, a known medical history of hypercholesterolemia or total serum cholesterol > 6.5 mmol/l in the past 5 years), current smoking (defined as smoking during the last 6 months), Glasgow coma scale (GCS) score at admission, as well as the unadjusted in-hospital mortality. Additionally, we recorded antithrombotic treatment (Antiplatelet therapy, DOACs, VKA, Heparin/Low-Molecular-Weight Heparin (LMWH), or combinations) at the time of admission. Oral anticoagulants-related ICH was defined as an ICH in a patient using VKA or DOAC. Primary brain CT-scans were evaluated. Hematoma location was recorded as lobar, deep, infratentorial, or multifocal. The ICH volume was calculated using the validated ABC/2 formula.^22^ Finally, the presence of intraventricular extension was registered.

Data on the number of adult inhabitants in South-Limburg during both study periods were retrieved from the Dutch national statistics institute.^23^ Data on the total number of extramural oral anticoagulation-users (both VKAs and DOACs) in the Netherlands in 2008 and 2018 were retrieved from the Dutch Foundation for Pharmaceutical Statistics (SFK).

### Data analysis

Differences in patient characteristics between 2007–2009 and 2017–2019 for all ICH and OAC-related ICH were tested using Chi-square test for categorical and ordinal variables. For continuous variables, the Mann–Whitney U test was used. Statistical analyses were performed using SPSS version 25.0.

The crude annual incidence rates of ICH and OAC-related ICH for the study periods 2007–2009 and 2017–2019 were calculated by dividing the number of (OAC-related) ICH by the total person-years at risk for ICH. Person-years at risk in each study period were calculated by adding up the average inhabitant numbers of South-Limburg in the study years. Furthermore, we calculated the crude incidence rates for different age groups, and the total incidence rate age-adjusted to the Dutch and European population of 2008 and 2018, respectively.^24^ For all rates, the 95% Poisson confidence intervals (95%-CI) were calculated using Stata version 14.1. Finally, we calculated the age-strata-adjusted incidence rate ratios (IRR) and 95%-CI, by dividing the incidence rate of 2017–2019 for each age group by the incidence rate of 2007–2009 for each age group, for both total and OAC-related ICH, using Open Source Epidemiologic Statistics for Public Health version 3.01.^25^

## Results

We included a total of 652 ICH cases from 2007–2009 and 522 ICH cases from 2017–2019. Results from 2007–2009 were previously described and were recalculated in this study.^21^

Clinical and radiological characteristics for both study periods are described in Table 1. In 2007–2009, 168 (25.8%) patients used OAC (all VKAs). In 2017–2019, 121 (23.2%) patients used OAC, of which 70 (57.9%) used VKAs and 51 (42.1%) used DOACs. Intracerebral hemorrhage patients of the 2007–2009 cohort used significantly more often antiplatelet drugs, were more often smokers, had a worse GCS-score at admission, a larger ICH volume, more often intraventricular extension, as well as a significantly higher in-hospital mortality.

**Table 1.** Patient characteristics of all adult ICHs from 2007–2009 and 2017–2019.

	N	All ICHs 2007–2009	All ICHs 2017–2019	*p* value^a^
N	1174	652	522	
Sex, men (%)		339 (52.0)	262 (50.2)	.539
Age, median in y [IQR]		76.3 (66.6–83.0)	75.0 (65.9–82.7)	.372
OAC-related ICH (%)		168 (25.8)	121 (23.2)	.307
VKA-related ICH (%)		168 (25.8)	70 (13.4)	<.001
DOAC-related		0	51	<.001
Prior ICH (%)	1168	65 (10.0)	56 (10.8)	.695
Antiplatelet therapy (%)	1163	228 (35.5)	146 (28.0)	.007
Hypertension (%)	1152	431 (68.3)	348 (66.8)	.586
Diabetes mellitus (%)	1157	110 (17.3)	100 (19.2)	.405
Hypercholesterolemia (%)	1148	260 (41.5)	234 (44.8)	.262
Current smoking (%)	835	113 (27.4)	79 (18.7)	.003
GCS-score ≤ 8 (%)	1173	134 (20.6)	75 (14.4)	.006
ICH location (%)				
Lobar		290 (44.5)	240 (46.0)	
Deep lobar		272 (41.7)	222 (42.5)	.027
Infratentorial		90 (13.8)	55 (10.5)	
Multifocal		0	5 (1.0)	
ICH volume, median in cc [IQR]	1165	15.6 [4.3–44.5]	11.9 [3.5–33.0]	.023
Intraventricular extension (%)		286 (43.9)	172 (33.0)	<.001
Hospital mortality (%)		256 (39.3)	160 (30.7)	.002

^a^*p* value for the difference between All ICHs 2007–2009 and All ICHs 2017–2019.

Abbreviations: ICH - intracerebral hemorrhage; *N* - number of ICH cases; y - years; IQR - interquartile range; OAC-related ICH - oral anticoagulant-related intracerebral hemorrhage; VKA-related ICH - Vitamin K-related intracerebral hemorrhage; DOAC-related ICH - Direct Oral Anticoagulant-related intracerebral hemorrhage, GCS-score - Glasgow Coma Scale-score; cc - cubic centimeter.

Differences in clinical and radiological characteristics between OAC-related ICH cases of the two cohorts are presented in Table 2. In the 2007–2009 cohort, more patients had a history of prior ICH, patients used significantly more often antiplatelet drugs in addition to OAC, more often had hypertension, had a worse GCS-score at admission, more frequent intraventricular hemorrhagic extension, as well as a significantly higher in-hospital mortality. We also compared characteristics of VKA-related ICH patients from 2007–2009 to VKA-related ICH patients from the 2017–2019 period, as well as the DOAC-related ICH patients to the VKA-related ICH patients from the 2017–2019 period. These results can be found in supplementary Table 1.

**Table 2.** Patient characteristics of OAC-related ICHs from 2007–2009 and 2017–2019.

	N	OAC-ICH 2007–2009	OAC-ICH 2017–2019	*p* value
N	289	168	121	
Sex, men (%)		88 (52.4)	60 (49.6)	.639
Age, median in y [IQR]		79.1 [72.6–84.2]	80.2 [72.7–86.4]	.209
Prior ICH (%)	288	16 (9.6)	4 (3.3)	.039
VKA-related ICH (%)		168	70	<.001
DOAC-related ICH		0	51	<.001
Antiplatelet therapy (%)		14 (8.3)	3 (2.5)	.037
Hypertension (%)	285	148 (90.2)	91 (75.2)	.001
Diabetes mellitus (%)	283	37 (22.8)	24 (19.8)	.543
Hypercholesterolemia (%)	284	83 (50.9)	63 (52.1)	.848
Current smoking (%)	206	21 (19.8)	18 (18.0)	.740
GCS ≤ 8 (%)	288	46 (27.4)	17 (14.2)	.007
ICH location (%)				
Lobar		70 (41.7)	45 (37.2)	
Deep lobar		63 (37.5)	58 (47.9)	.056
Infratentorial		35 (14.3)	16 (10.7)	
Multifocal		0	2 (1.7)	
ICH volume, median in cc [IQR]	285	17.8 [5.2–62.0]	13.3 [3.9–32.6]	.106
Intraventricular extension (%)		91 (54.2)	45 (37.8)	.004
Hospital mortality (%)		92 (54.8)	47 (38.9)	.008

^a^*p* value for the difference between All ICHs 2007–2009 and All ICHs 2017–2019.

Abbreviations: ICH - intracerebral hemorrhage; OAC-ICH - oral anticoagulant-related ICH, *N* - number of OAC-ICH cases; y - years; IQR - interquartile range; VKA-related ICH - Vitamin K-related intracerebral hemorrhage; DOAC-related ICH - Direct Oral Anticoagulant-related intracerebral hemorrhage, GCS-score - Glasgow Coma Scale-score; cc - cubic centimeter.

### Incidence of total ICH

For both study periods, the crude ICH incidence as well as the Dutch and European age-adjusted ICH incidence rates is listed in Table 3. In 2007–2009, the crude incidence of total ICH was 40.9 per 100,000 person-years (95%-CI: 37.8–44.1). In 2017–2019, the crude incidence of total ICH was significantly lower, being 32.4 per 100,000 person-years (95%-CI: 29.7–35.3). The IRR for total ICH is 0.67 (95%-CI: 0.60–0.75) (Table 3). The adult population of South-Limburg, the Netherlands, has increased in number and notably aged in the past decade.

**Table 3.** Incidence rates per (100,000 person-years) of all adult (OAC-related) ICHs from 2007–2009 and 2017–2019.

Age groups	2007–2009					2017–2019				
	ICH	OAC-ICH				ICH	OAC-ICH			
	N	N	Population at Risk (person-years)	Incidence rate all ICHs (95%-CI)	Incidence rate OAC-ICH (95%-CI)	N	N	Population at Risk (person-years)	Incidence rate all ICHs (95%-CI)	Incidence rate OAC-ICH (95%-CI)
18–44 years	20	12	628,438	3.2 (1.9–4.9)	1.0 (0.5–1.7)	11	11	574,475	1.9 (1.0–3.4)	1.0 (0.5–1.7)
45–54 years	31		315,607	9.8 (6.7–13.9)		35		270,446	12.9 (9.0–18.0)	
55–64 years	96		285,791	33.6 (27.2–41.0)		76		301,765	25.2 (19.8–31.5)	
65–74 years	139	40	199,670	69.6 (58.5–82.2)	20.0 (14.3–27.3)	138	22	257,008	53.7 (45.1–63.4)	8.6 (5.4–13.0)
75–84 years	260	81	126,912	204.9 (180.7–231.3)	63.8 (50.7–79.3)	173	51	151,083	114.5 (98.1–132.9)	33.8 (25.1–44.4)
>85 years	106	35	39,384	269.1 (220.4–325.5)	88.9 (61.9–123.6)	89	37	57,061	156.0 (125.3–191.9)	64.8 (45.7–89.4)
**Crude (all ages)**	652	168	1,595,800	**40.9 (37.8–44.1)**	**10.5 (9.0–12.3)**	522	121	1,611,838	**32.4 (29.7–35.3)**	**7.5 (6.2–9.0)**
**Age-adjusted rate NL**				**34.8 (32.0–37.8)**	**8.8 (7.4–10.4)**				**27.1 (24.6–29.8)**	**6.1 (4.9–7.4)**
**Age-adjusted rate EU**				**37.6 (34.7–40.7)**	**9.9 (8.4–11.6)**				**28.6 (26.1–31.3)**	**6.8 (5.6–8.2)**

Bold summarize all age categories.

### The incidence of OAC-related ICH

The crude ICH incidence as well as the Dutch and European age-adjusted OAC-related ICH incidence rates is listed in Table 3. In 2007–2009, 25.8% of all adult ICHs were OAC-related. The crude incidence of OAC-related ICH was 10.5 per 100,000 person-years (95%-CI: 9.0–12.3). In 2017–2019, 23.2% of all adult ICHs were OAC-related. The crude incidence of OAC-related ICH was significantly lower compared to the 2007–2009 cohort, being 7.5 per 100,000 person-years (95%-CI: 6.2–9.0). The IRR for OAC-related ICH was 0.58 (95%-CI: 0.46–0.73) (Table 4).

**Table 4.** Incidence rate ratios (IRR) for (OAC-related) ICH in the 2017–2019 cohort, with the 2007–2009 cohort serving as reference.

Age groups	IRR all ICHs (95%-CI)	IRR OAC-ICH (95%-CI)	IRR Non-OAC-ICH (95%-CI)
18–44 years	0.60 (0.29–1.26)	0.98 (0.43–2.22)	0.88 (0.69–1.13)
45–54 years	1.32 (0.81–2.14)		
55–64 years	0.75 (0.56–1.01)		
65–74 years	0.77 (0.61–0.98)	0.43 (0.25–0.72)	0.91 (0.70–1.19)
75–84 years	0.56 (0.46–0.68)	0.53 (0.37–0.75)	0.57 (0.46–0.72)
>85 years	0.58 (0.44–0.77)	0.73 (0.46–1.16)	0.51 (0.35–0.72)
Total (age-adjusted)	0.67 (0.60–0.75)	0.58 (0.46–0.73)	0.71 (0.62–0.81)

### Anticoagulation users

The total number of OAC-users in the Netherlands increased from 352,001 in 2008 to 573,073 in 2018. In 2008 only VKAs were used as OAC, in 2018 there were 312,012 (54.5%) VKA-users and 277,077 (48.4%) DOAC-users.^26^

## Discussion

This study shows a significant decrease in both total ICH incidence and OAC-related ICH incidence in South-Limburg, the Netherlands, in the past decade. At the same time, the number of OAC-users has notably increased by approximately 63% in the Netherlands in recent years, with a shift from VKAs to DOACs.

The Dutch population age-adjusted total ICH incidence in our study is higher when compared to the ICH incidence of 20.7 per 100,000 person-years as reported in the Dutch population study of Jolink et al.^1^ in 2015. In other studies, ICH incidence rates range from 10.0 to 46.0 per 100,000 person-years.^6,8–12,14,16,27–29^ However, we determined the adult incidence rate, while some studies report life-time incidence, which leads to lower incidence rates; yet other studies report incidence in an elderly population, which will result in higher incidence rates. Furthermore, the region South-Limburg is known for its lower socioeconomic and health status compared to other regions in the Netherlands, which may result in a higher incidence.^30^

The total ICH incidence decreased in the past decade by approximately 33%. This reduction is mainly seen in older age groups. Some studies show similar results,^11,27^ though most studies report a stable or even increased incidence rate in the older population.^8–10,14,16^ A possible explanation for a decreased ICH incidence in our study is the further improvement of cardiovascular risk management, in particular antihypertensive management, and less smoking. Favorably, ICHs were smaller and had lower in-hospital mortality in the 2017–2019 cohort compared to the 2007–2009 cohort.

The proportion of approximately 25% OAC-related ICHs in both study periods is relatively high compared to some other studies, which show proportions ranging from 5% to 25%.^9,14,17,19,31,32^ Furthermore, previous studies show that the proportion of OAC-related ICH seems to have risen in recent years, yet it remained stable in our study.^10,18–20^

In the Netherlands, the number of OAC-users has increased by approximately 63% in the past decade.^26^ Since DOACs are available, their use has risen exponentially and now exceeds that of VKAs.^3^ Nevertheless, the OAC-related ICH incidence in our study shows an overall reduction of approximately 42% in the past decade (Table 3). Moreover, VKA-related ICH incidence in our study shows an overall reduction of about 67% (supplemental Table 2), even though the number of VKA-users remained relatively stable. Possible explanations for the decrease in OAC-related ICH incidence might be the increased proportional use of DOACs (carrying a lower ICH risk) and further improvement of cardiovascular risk management (especially antihypertensive management). Additionally, those VKA users having labile international normalized ratio (INR) and thereby an increased risk of major bleeding events including ICH^33^ have likely been switched from VKA to DOAC. This is supported by our finding that INR values of VKA-related ICH patients from the 2007–2009 period were significantly higher on admission and more often supratherapeutic, when compared to VKA-related ICH patients from the 2017–2019 period. Lastly, the number of OAC-related ICH patients with a prior ICH was significantly higher in the 2007–2009 cohort when compared to the 2017–2019 cohort, as were the number of patients who simultaneously used OAC and antiplatelet drugs. Both the resumption of OAC after ICH and the concurrent use of OAC and antiplatelet agents might increase the risk for recurrent ICH,^34,35^ and these prescription pattern of clinicians could have changed over time.

In contrast to our findings, a recent Spanish study showed a threefold increase in OAC-related ICH incidence from 2008 to 2015.^19^ However, their first study period was only 4 months, which was extrapolated to yearly incidence. Also, their study population varied between the different time periods, and whether anticoagulation prescription rates changed is not known. A single other recent study, performed in Denmark between 2010 and 2017, showed an increased number and proportion of OAC-ICH events as well as increase of OAC use over time, despite a stable total number of ICH events.^20^These findings are in contrast to our findings. The discrepancy in the proportion of OAC-ICH patients at the study start, being 25.8% in our study and 12% in the Danish study, likely reflects a differing study population or baseline prescription rate of OAC, which could explain these conflicting findings.

Our study has limitations. First, this study is hospital-based. Patients who were hospitalized outside the inclusion region or who were not seen in the hospital may have been missed. Still, our study region is well-demarcated, hospital care is nearby and easily accessible, and referral from general practitioners is immediate, in compliance with regional guidelines. Hence, we expect the number of missing patients to be limited. Due to the retrospective design of the study, we were unable to verify OAC compliance of the included patients. Direct Oral Anticoagulant plasma concentrations were not routinely obtained, which precludes the assessment of compliance and therapeutic range of DOAC-users. Oral anticoagulant prescription rates were only available on national basis, and therefore not specified for the catchment area. Finally, our study region is known for its poorer socioeconomic and health status, possibly leading to an overestimation of ICH incidence when extrapolating to national level.^21,30^

In conclusion, the total ICH and OAC-related ICH incidence decreased over the past decade in South-Limburg, the Netherlands, despite the aging population and increasing number of OAC-users. The introduction of DOACs, and possibly an improved cardiovascular risk management and change in OAC prescription pattern, could explain these findings.
